# Low-Roughness-Surface Additive Manufacturing of Metal-Wire Feeding with Small Power

**DOI:** 10.3390/ma14154265

**Published:** 2021-07-30

**Authors:** Bobo Li, Bowen Wang, Greg Zhu, Lijuan Zhang, Bingheng Lu

**Affiliations:** 1 School of Mechanical Engineering, Xi’an Jiaotong University, Xi’an 710049, China; boboli@stu.xjtu.edu.cn (B.L.); wangbowen@stu.xjtu.edu.cn (B.W.); 2 National Innovation Institute of Additive Manufacturing, No. 997, Shanglinyuan 8th Road, Gaoxin District, Xi’an 710300, China; zhanglj@sdut.edu.cn

**Keywords:** additive manufacturing, surface roughness, direct metal deposition, wire feed, fine metal wire, laser and Joule heating

## Abstract

Aiming at handling the contradiction between power constraint of on-orbit manufacturing and the high energy input requirement of metal additive manufacturing (AM), this paper presents an AM process based on small-power metal fine wire feed, which produces thin-wall structures of height-to-width ratio up to 40 with core-forming power only about 50 W. In this process, thermal resistance was introduced to optimize the gradient parameters which greatly reduces the step effect of the typical AM process, succeeded in the surface roughness (*Ra*) less than 5 μm, comparable with that obtained by selective laser melting (SLM). After a 10 min electrolyte-plasma process, the roughness of the fabricated specimen was further reduced to 0.4 μm, without defects such as pores and cracks observed. The ultimate tensile strength of the specimens measured about 500 MPa, the relative density was 99.37, and the Vickers hardness was homogeneous. The results show that the proposed laser-Joule wire feed-direct metal deposition process (LJWF-DMD) is a very attractive solution for metal AM of high surface quality parts, particularly suitable for rapid prototyping for on-orbit AM in space.

## 1. Introduction

In recent years, metal additive manufacturing gradually developed into a new strategic manufacturing technology, which plays an important role in the aerospace manufacturing industry [1,2,3,4]. In metal additive manufacturing (AM), raw materials include metal powder and metal wires. Currently, metal material manufacturing technology research mainly focuses on powder-based process and wire-feed (WF)-based process. The powder-based process was first widely developed and applied in manufacturing. Its representative technology is selective laser melting (SLM) [5,6]. SLM was the first developed, and after improvements, is most widely used in sophisticated fields such as aerospace and medical engineering. The SLM is mature and has high printing precision, because of the use of small powder sizes (15–40 μm) and optimization of process parameters that enable the surface roughness of parts to be reduced significantly, to 6–20 μm for SLM techniques [7,8]. However, it has disadvantages, e.g., expensive raw materials, the lower utilization efficiency of materials (20–30%), and poor density [9,10] and so on. More importantly, the SLM cannot be used in a microgravity environment where the motion of the powdered particles is beyond control.

Hence, some researchers shifted their focus to wire feed techniques, which, when compared to SLM, has lower cost of raw materials, higher utilization efficiency (nearly 100%), and lower impact on the environment [11,12,13]. The weakness of wire-feed-based AM is that its surface quality is much lower than that obtained by SLM. In most of the reported research works, metal wires of a diameter of about 1 mm are used, and the width of deposition is 5 to 10 times the diameter of the wire [14,15]. Using SLM, the layer height and thickness varies between 5 and 19 mm, requiring a considerable amount of subtractive post-process, when used to fabricate high-finish parts.

The roughness is a significant index for evaluating AM parts. The lower roughness causes easier fitting, more erosion-resistance, and better sealing; thus, alleviating wear-out and prolonging service life [16,17,18]. Moreover, with lower roughness, the burden of the post-process is reduced greatly, and some AM parts can even be directly fitted. Lower roughness can further save the material loss by subtractive post-process. There are three factors that make the SLM obtain lower roughness: small size of powder, high-precision heat source, and process parameter optimization. Aiming at these three factors, the study was improved correspondingly.

Currently, the commonly used heat sources include plasma, electric arc, electron beam, laser and compound heat sources [19,20]. To fabricate high-finish parts, laser heating is the first choice for heat source. When well-focused, the light spot of the laser is tiny, the heating-power per unit area is very high, and the energy is released intensely. However, the electro-thermal conversion rate of laser is rather low, thus requiring a large-power laser device for metal AM. Demir AG al. [21] used a fine-wire AM technique to build thin-wall structures with aspect ratios up to 20, with a 301 stainless steel 0.5 mm wire as the raw material and a pulse laser heat source of peak power 5 KW. Shaikh MO et al. [22], using 0.1 mm stainless steel wires and a pulse laser, 6 KW peak power, fabricated a metal part with a roughness (*Ra*) of 8–16 μm. These two studies demonstrated that laser-heated fine-wire AM can be used to produce thin-walled parts with good surface morphology. However, these fine wire AM need large laser-power.

Most studies have focused on increasing the deposition efficiency and cost performance [23,24], and less attentions have been focused on obtaining good AM morphology under limitation of input of small heat power. Therefore, to yield high precision metal for on-orbit manufacturing, the crucial issue is to solve the power deficiency of small single laser.

A fine-wire-based direct metal deposition (DMD) technique was developed in this study, using a 0.3 mm-diameter 316 stainless steel wire as the raw material and laser and Joule heating as a compound heat source, process parameters were adjusting layer-by-layer. When Joule heating wire during processing, it can greatly reduce the power requirement of laser, thus the total heat input was controlled and the heat in the forming process was effectively managed. Using this method, 316 L thin-walled specimens of an aspect ratio up to 40 were successfully fabricated. The surface roughness, microstructure, density, hardness, and mechanical properties of specimens were tested, and the main factors affecting the surface roughness were analyzed. It is expected that, with its small size and light weight, the laser-Joule wire feed-direct metal deposition (LJWF-DMD) equipment is a premium solution for in situ manufacturing in space.

## 2. Materials and Methods

### 2.1. Materials

Both the metal wire and the baseplate used in this study were fabricated of 316 L stainless steel. The mass percent of the steel is shown in Table 1.

### 2.2. Experimental Design and Setup

**Figure 1:**
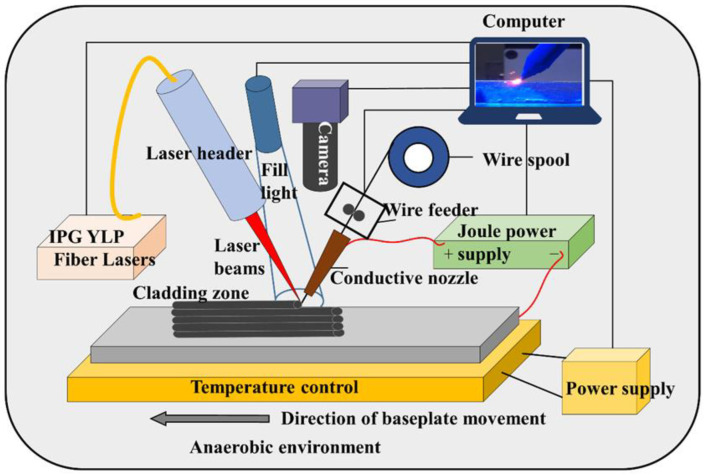
Schematic of the LJWF-DMD AM system.

An experimental platform based on laser-Joule wire feed-direct metal deposition (LJWF-DMD) was built in this study. Figure 1 is a schematic of the platform, which consisted of a laser unit with a laser head (IPG, Oxford, MA, USA), a camera with a fill lamp(Helica, Shenzhen, China), a wire feeder, a motion platform, a Joule heating power supply and a baseplate preheating temperature control unit. Laser and Joule heat were used as the compound heat source in the printing process, and the baseplate preheating unit provided a stable initial temperature. Oxidization during the printing process was prevented by placing the entire platform in a DELLIX standard glove box ((DELLIX, Chengdu, China) in which the oxygen content and moisture content were both below 10 ppm.

**Figure 2:**
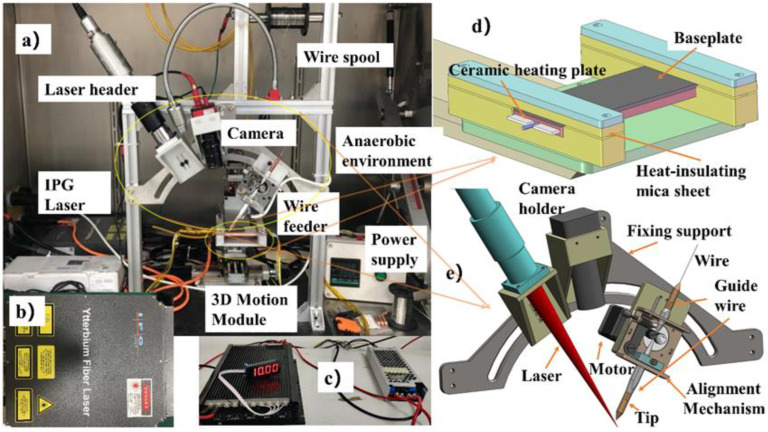
Details of the LJWF-DMD AM system. (**a**) Overview of test equipment. (**b**) Laser device. (**c**) Joule heating power supply. (**d**) Baseplate temperature control system. (**e**) Laser-wire alignment adjustment mechanism.

A photo of the experiment device is shown in Figure 2a. A laser head, wire feeder and camera are mounted on the sliding blocks of the fixture. The framework is fabricated of aluminum. The base is a porous optical slab that is easy to install and adjust. A YLP-series fiber laser (IPG, MassachusettsdUSA) is one of the major heat sources for FW-DMD manufacturing. In Figure 2b is the small (360 mm × 300 mm × 110 mm) and light (5 kg) laser unit, which is equipped with an independent air-cooling system. After collimation and focusing, the focal length of the laser header is 250 mm and the diameter of the focal spot is 80 μm. Since the wire diameter is 300 μm, the laser spot should be negatively defocused to 350 μm so that the laser spot can cover the wire fully with adequate energy density under the limited power of 50 W only. A Joule heating source was used mainly for melting wire. In Figure 2c, the power supply of the Joule heating source is a customized current source with an adjustable range of 0–30 A and a power less than 50 W. Three-dimensional (3D) movement of the baseplate is realized by means of a ball-screw three-axis motion platform with a STEVAL-3DP001V1 (STMicroelectronics, Geneva, Switzerland) main control unit. A stable high-temperature field was created on the baseplate by using a pre-heating unit consisting of ceramic heating elements, heat-isolating mica sheets and a proportional-integral-derivative (PID) temperature-control power supply, as shown in Figure 2d, which can provide a maximum heating temperature of 600 °C and can be real-time monitored and controlled to maintain the temperature at a constant level. To enhance image resolution, the laser welding camera is equipped with a blue-light fill lamp for tracking the laser-wire and observing the printing areas in real time. A short-range wire delivery system was designed to feed fine wire of 0.3 mm in diameter smoothly, and suppress the disturbances and ensure accurate aligning of the laser beam with the fine wire. This system is comprised of a motor with drive, a wire guide and laser-wire alignment adjustment mechanisms. The abovementioned laser header, wire feeder and camera were mounted on the sliding blocks of an arc bracket to facilitate implementation of experiments from different orientations (Figure 2e).

Table 2 gives main the parameters and adjustment ranges, which, determined through a lot of experiments, include the laser power (*P_L_*), Joule heating power (*P_J_*), laser incident angle (*A*_1_), wire feeding angle (*A*_2_), wire feeding speed (*V_W_*) and moving speed (*V_M_*) required to ensure AM stability. A key parameter (*K*) was defined as the ratio of wire feeding speed to moving speed, i.e., the equation:

Experiments were carried out for single-layer and multi-layer depositions. In view of different heating effects under different thermal environments, the parameters were progressively changed for a different number of layers. Studies on the influence of the wire feed direction on printing quality [25,26,27] already showed that front wire feeding produces parts with the highest surface smoothness. Hence, front wire feeding was adopted in this study.
Water content≤10 ppmMaximum Joule power 50 WBaseplate temperatures≤600 ℃

The maximum optical power during actual printing was measured to be 50 W on a PRIMES Cube laser power meter.

### 2.3. Characterization

The surface roughness of a thin-walled specimen was measured mechanically and optically using a MarSurf M300C roughness meter and a Smartproof 5 laser scanning confocal microscope (Zeiss, Jena, Germany), respectively, according to ISO4287. The dimensions of the multi-layer thin-walled structure were measured using an NSCING Vernier caliper (NscingEs, Shuzhou, China) and a digital micrometer with an accuracy of 0.001 mm. The Vickers hardness of sections of the thin-walled part at different heights was measured using a Mitutoyo HM-200 automatic Vickers hardness tester (Mitutoyo, Sanfeng, Japan) 10 s after the application of a 0.2-N load. A universal material testing machine (INSTRON 5982, Norwood, MA, USA) was used to test the tensile strength and elongation at the fracture of the specimens. The specimens fractured by tensile failure were imaged using a JSM-7900F field emission scanning electron microscope (JEOL, Tokyo, Japan) provided by JEOL. A mud saw and abrasive paper were used to prepare multi-layer single-track thin-walled cross-sections for imaging with an Axio Ver.A1 inverted metallurgic microscope (Zeiss, Jena, Germany). The image processing software ImageJ was used to measure the widths, heights, sectional areas and wetting angles of the cross-sections. The density of the thin-walled part was measured using a DahoMeter DH-220MN (Dahometer, Shenzhen, China) electronic density and specific gravity tester based on the Archimedes method of water displacement; the buoyancy and volume of an object in water are equal in magnitude to the weight and volume of water displaced, respectively. Hence, the density of the specimen is obtained as follows:

(2) $$m_{w}=m_{1}-m_{2}$$

(3) $$V_{w}=\frac{m_{w}}{\rho_{w}}$$

(4) $$\rho_{1}=\frac{m_{1}}{V_{w}}=\frac{m_{1}}{\frac{m_{1}-m_{2}}{\rho_{w}}}=\rho_{w}\frac{m_{1}}{m_{1}-m_{2}}$$

where *m_w_* is the mass of water displaced by the tested specimen (g); *m*_1_ is the specimen mass in air (g); *m*_2_ is the specimen mass in water (g); *ρ_w_* is the density of water (g·cm^−3^); *V_w_* is the volume of water displaced by the specimen (cm^3^); and *ρ*_1_ is the specimen density (g·cm^−3^).

## 3. Results

### 3.1. Single-Layer Deposition

**Figure 3:**
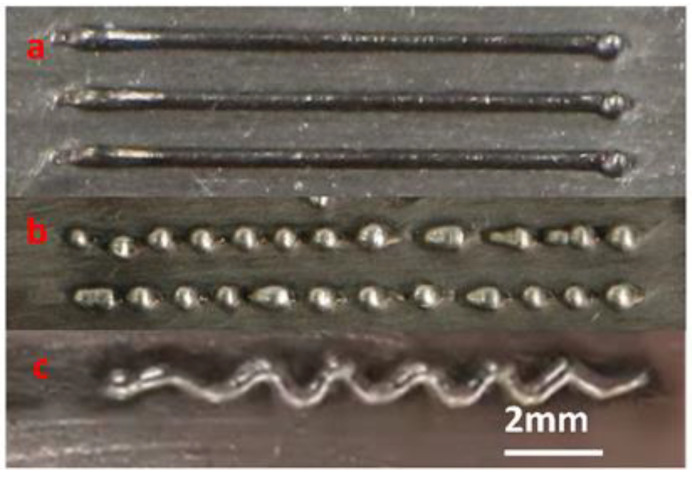
Effect of different parameters on the morphology of specimens in single-track LJWF-DMD AM. (**a**) Successful single track. (**b**) Discontinuity and spheroidization. (**c**) Wire bending and deposition.

The main factors that affected the printing quality during single-track formation were *P_L_*, *P_J_*, *A*_1_, *A*_2_, *V_W_* and *V_M_*, see Figure 3. Figure 3a shows that the well-matched input energy and wire feeding produces good printing quality. On the other hand, excessive energy input and low feed speed resulted in premature wire melting, where the wire melted into small balls before entering the molten pool and the printing process was disrupted as Figure 3b indicates. Figure 3c shows that insufficient power input, higher feed speed or lower moving rate (larger *K*) caused the wire to bend and deposit, resulting in the failure of the process.

**Figure 4:**
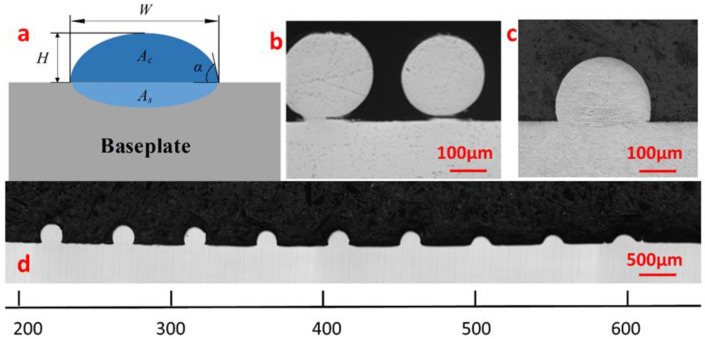
LJWF-DMD single-track cross-sections. (**a**) Geometry of the single-track section. (**b**) Single-track sections obtained using a single-laser heat source. (**c**) Single-track section obtained using a laser and Joule heating. (**d**) Single-track sections obtained at different baseplate heating temperatures.

If the input energy is too low, interlayer bonding cannot be achieved even though the wire melts. It should be noted that the temperature of the baseplate also affects the quality. The specimen cross-section was analyzed to assess the single-track part quality. Figure 4a shows a typical single-track section. The influential parameters include the single-track width (W), single-track height (H), printing area (Ac), molten area (As) and wetting angle (α), and dilution rate (D) which is defined as As/(Ac + As). The α should be maintained as small as possible to facilitate good overlap between multiple single tracks during transverse splicing to prevent pore formation during printing.

The single tracks formed using various parameters were cross-sectioned and fabricated into specimens. The sectional morphology of the polished specimens was observed by an optical microscope to check for defects. Comparisons were observed between the single-track sections formed using compound heat sources and those using only single laser. Given *P_L_ =* 50 W, *V_W_* = 15 mm/min and *V_M_* = 15 mm/min, using single laser, the left track has a very small contact area with the baseplate, and the right one does not contact at all, as shown in Figure 4b, i.e., insufficient energy results in poor bonding between the wire and baseplate. Using the same parameters, after introducing a Joule heating current *I* = 10 A and raising the temperature of the baseplate to 300 °C, tracks bond well with the baseplate, without defects such as pores and cracks, as shown in Figure 4c. Figure 4d shows the variation of the sectional morphology of specimens with the baseplate temperature increasing and thus decreasing the thermal gradient between the baseplate and the molten pool can effectively improve the spreading of the single track: both width (W), and dilution (D), increase, whereas height (H), and wetting angle (α), decrease. When α is below 90°, its section is smaller than a semicircle, and the single-track can be used for transverse multi-pass splicing.

### 3.2. Thin-Wall Deposition

**Figure 5:**
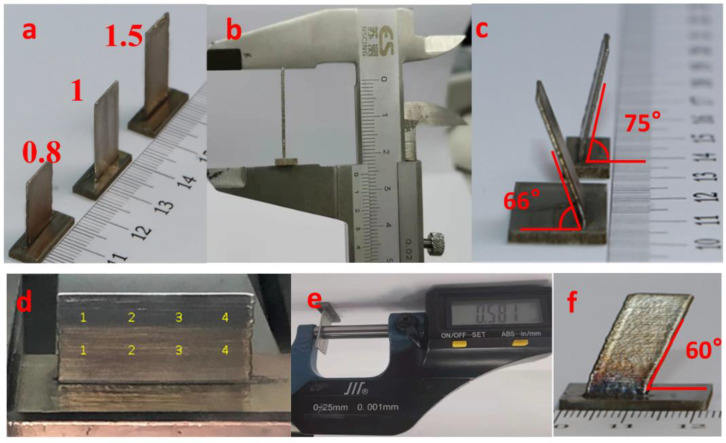
LJWF-DMD thin-walled parts. (**a**) Parts formed using various values of *K*. (**b**) Height measurement. (**c**) Thin-walled part inclined towards the *Y*-axis. (**d**) Measuring points. (**e**) Thickness measurement. (**f**) Thin-walled part inclined towards the *X*-axis.

To examine whether the proposed technique is valid, thin-walled specimens were prepared on a 2 mm-thick baseplate using a laser power of 50 W, Joule heating current of 10 A and a baseplate temperature of 500 °C (Figure 5a). Parameters *V_W_* and *V_M_* were adjusted according to the number of the layers. Figure 5a shows three 150-layer thin-walled parts corresponding to different *K* (0.8, 1 and 1.5). The specimen dimensions were measured. The 150-layer deposition was 24 mm high at *K* = 1.5 (Figure 5b). The thickness of the specimen was measured at different points, being 0.589 mm on average. Its aspect ratio reached over 40. In Figure 5d, the thickness was measured at eight points, which are equally spaced at the upper and lower portion of a wider thin-wall. The measurements are given in Table 3, with the average wall thickness 0.581 mm, average deviation 0.017 mm, and range 0.047 mm. These results show the specimens thus formed using laser plus Joule heating with a uniform thickness and good dimensional stability. Figure 5c,f show that the formation of specimens can be performed with different inclination angles to the *X*- and *Y*-axes, respectively.

### 3.3. Surface Finish

**Figure 6:**
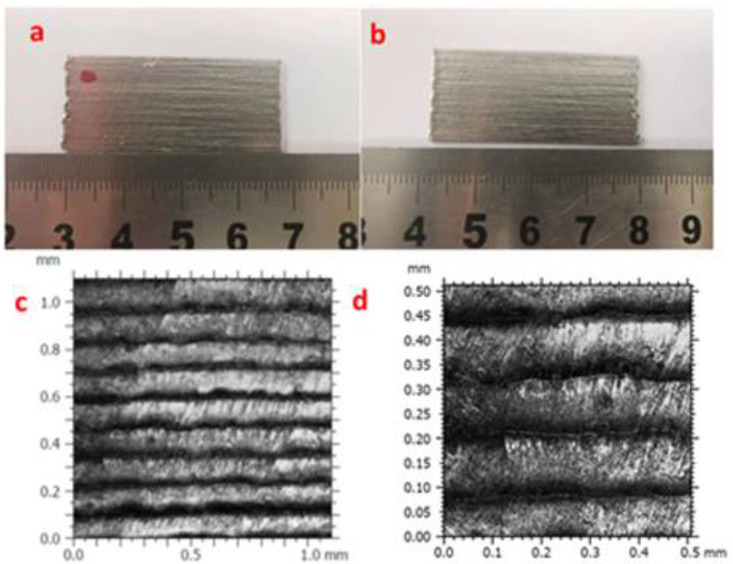
Surface morphology of LJWF-DMD-printed specimens, (**a**) front view, (**b**) back view, (**c**) 2D optical microscopy stair-step effect image (1) (**d**) and enlarged 2D optical microscopy stair-step effect image (2).

Surface roughness is one of the most important characteristics of AM parts. Many interdependent factors and parameters have significant influence on the surface roughness. The average surface roughness (*Ra*), is the mean variation in distance of a surface profile to the measurement center line, and the maximum deviation (*Rz*), is the distance between the highest and lowest points on the contour [28]. These two indexes, *Ra* and *Rz*, are the most commonly used indicators of surface roughness. It is known that layer-by-layer material addition creates an inherent stair-step effect on the test specimen surface. Figure 6a,b show that both the front (marked by a red dot) and back surfaces have the same morphology. A two-dimensional (2D) optical microscopy image of the stair-step effect under a 20× objective is given in Figure 6c, and its enlarged optical microscopy image in Figure 6d. The image shows clearly the lines of deposition layers on the thin-walled part. The contour is cyclical with the height of each layer, approximately 0.11 mm. According to measurement specifications, the corresponding sampling length is 1 mm. As the specimen surface is not perfectly even and the roughness varies at different points, a single sampling length could not indicate accurately the surface roughness as a whole. Therefore, multiple sampling lengths were used here at the front and back of the specimens. The *Ra* and *Rz* were measured for both the front and back sides of two 150-layer thin-walled parts formed at *K* = 1 and *K* = 1.5. Table 4 lists horizontal and vertical measurements at three points. It can be seen the roughness is very low in the horizontal direction, indicating printing was smoothly completed. On the other hand, in the vertical direction, the stair-step effect is significant and the roughness is much larger. It is noted that, with *K* increased, the stair-step effect and hence roughness become worse, although the printing efficiency improves.

**Figure 7:**
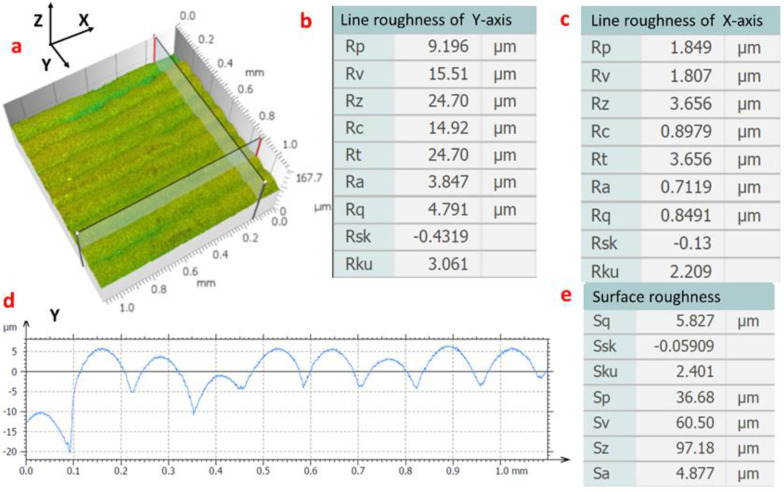
Surface roughness of LJWF-DMD thin-walled part. (**a**) 3D confocal microscopy images. (**b**) Line roughness along *Y*-axis. (**c**) Line roughness along *X*-axis. (**d**) Surface contour extracted in the printing direction. (**e**) Specimen surface roughness.

The 3D surface morphology of the thin-walled part formed at *K* = 1 was observed under a laser scanning confocal microscope and is shown in Figure 7a. Sections were extracted along the *X*- and *Y*-axes, i.e., in the horizontal and vertical (deposition) directions, respectively. The line roughness measured in both directions, with distortion corrected and noise removed, are given in Figure 7b,c. The values given by Smartproof 5 are close to those listed in Table 4, indicating the data are credible. Figure 7d shows the contour lines along the *Y*-axis (the printing direction) on the extracted surface. As shown, within the 1 mm range, nine crests and troughs are observed, corresponding to the stair-step effect caused by deposition layer by layer, thus making the contour vary cyclically from layer to layer. The surface roughness (in 3D) is measured and calculated based on ISO4287. The measurements are given in Figure 7e, including the surface arithmetic mean height (*Sa*), the maximum height (*Sz*), the root-mean-square (RMS) height (S*q*), the skewness (*Ssk*), the kurtosis (*Sku*), the peak (*Sp*), and the vale (*Sv*) [29]. These data demonstrate that the proposed laser and Joule-heating technique (LJWF-DMD) can effectively reduce the stair-step effect and improve the surface quality of thin-walled parts. For comparison, the data on the reduction in surface roughness by LJWF-DMD and by other processes are given in Table 5.

### 3.4. Hardness and Density

**Figure 8:**
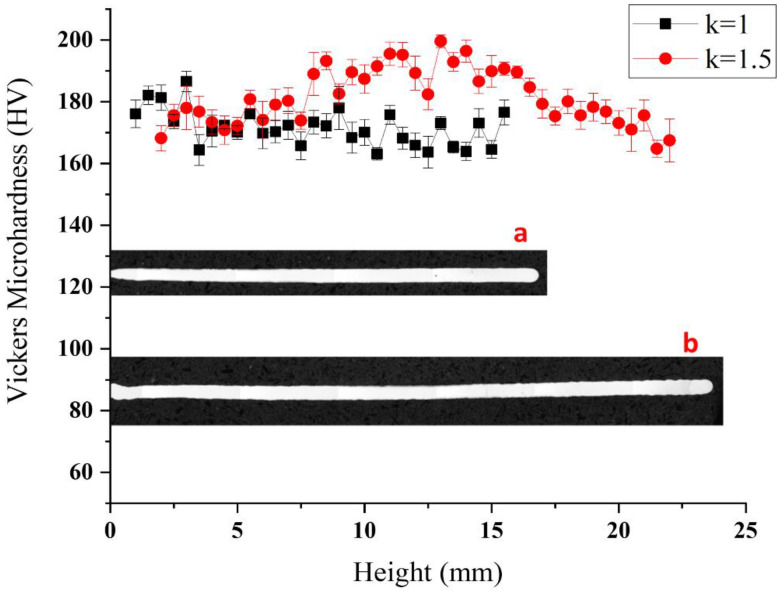
Hardness distribution at different heights of thin-walled parts with different *K*. Section of thin-walled part at (**a**) *K* = 1 and (**b**) *K* = 1.5.

The thin-walled parts formed at *K* = 1 and *K* = 1.5 were sectioned radially and fabricated into specimens. Figure 8a,b are optical micrographs of the sectional morphology of the polished specimens. The Vickers hardness was measured for sections at different heights of the thin-walled parts formed at *K* = 1 and *K* = 1.5. While having the same number of deposition layers (150), parts with different values of *K* have different heights. The values of hardness measured at 0.5 mm intervals are given in Figure 8. The hardness corresponding to *K* = 1 and *K* = 1.5 is approximately 170 and 180 HV, respectively, almost on a par with that of parts processed by WF AM [33]. The hardness is uniformly distributed along the height, with a small bump around the center of the height. Using laser plus Joule-heating DMD printing can also achieve competent and evenly-distributed hardness.

The mass of the test specimen, which is measured on the abovementioned Archimedes principle of water displacement (Equations (2)–(4)), was 4.9077 g and 4.2888 g in air and water, respectively. Given the water density of 1 g·cm^−3^, the density of the test specimen was calculated to be 7.93 g·cm^−3^. Compared to 316 L stainless steel with the standard value of density 7.98 g·cm^−3^, the relative density of the specimen formed using laser and Joule heating reached 99.37%, much better than that of the specimens produced by SLM AM [34].

### 3.5. Tensile Test

The tensile strength and elongation at fracture are two key indexes. The tensile strength is the stress when the part under test is subjected to the maximum plastic deformation. The elongation at fracture of a part is the ratio of its elongated length after fracture to its original length.

**Figure 9:**
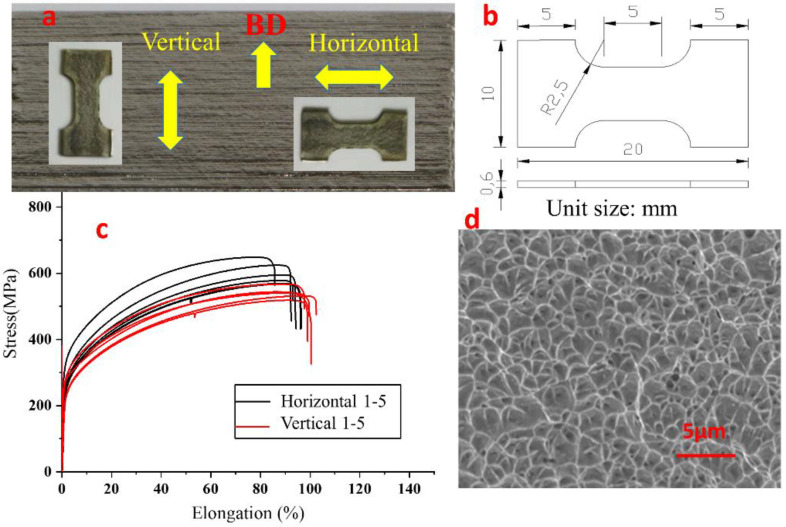
Tensile test and strength measurement of LJWF-DMD 316 L specimens. (**a**) Samples in vertical and horizontal directions. (**b**) Geometrical dimensions of the tensile specimen. (**c**) Stress/strain curves. (**d**) Scanning electron micrograph of fractures.

Tensile tests were conducted following ASTM E8, on a servo-electric INSTRON5982 frame equipped with an INSTRON 50-kN load cell, at the room temperature of 250 °C. Five samples were taken each in the vertical direction, along the building direction (BD), and horizontal direction, see Figure 9a. Samples, dog bone-shaped, were clamped by both ends, as shown in Figure 9b. The extension rate was 0.15 mm/min^−1^ and load-up was performed until the samples were fractured. The typical tensile properties of the thin-walled parts produced by LJWF-DMD are given in Figure 9c. The stress/strain curves show that the ultimate tensile strength (UTS) of horizontal samples reach to 612.2 ± 24.2 MPa, much higher than that of the vertical samples, 514.1 ± 17.1 MPa. In the respect of elongation to failure, however, horizontal samples are slightly weaker than vertical ones, 90.1 ± 5.9% vs. 98.2 ± 1.7%. It should be noted that even the lowest UTS, 514.1 ± 17.1 MPa, is comparable with that of the specimens fabricated of forged 316 stainless steel [35]. More significantly, the ductility or elongation to failure of the parts produced by using LJWF-DMD reaches 90% and over, superior to that of the parts using SLM AM [36,37]. In the scanning electron micrograph of the fracture shown in Figure 9d, there are a lot of fine even-sized dimples, with no defects such as cracks and pores observed.

### 3.6. Post-Processing

With low surface roughness obtained by using LJWF-DMD, the burden of post-processing of the formed parts can be reduced, at least avoiding using conventional cutting machining. For the post-processing of parts built by LJWF-DMD, electrolyte-plasma processing is sufficient and offers a variety of advantages, with no limitation on the shape of the parts, simultaneous processing of both exterior and interior, applicable to most metals, and high efficiency without requiring pre-treatment. In addition, during the post processing, there are no micro-cracks and residual stress generated because the part surface is subjected to only micro force. Therefore, electrolyte-plasma processing is particularly suitable for polishing parts with complex shapes, and it is a prosperous potential post-processing technique in AM [38].

In electrolyte-plasma processing, the specimen is the anode and the polishing solution and bath is the cathode. A voltage between 200 and 400 V is applied and controlled. The product from the chemical reaction adheres to the metal surface and is removed by using electric discharge. When the removal rate exceeds the production rate, polishing effect occurs. In this study, a 316 L thin-walled part printed by LJWF-DMD was put to electrolyte-plasma polishing, and the *Ra* value decreased from 3.6 μm to 0.4 μm for ten minutes. It is expected that the surface roughness could be decreased further if the polishing time is longer. The roughness of the AM parts after electrolyte-plasma processing are given in Table 6.

## 4. Discussion

### 4.1. Utilization of Joule Heating

A high-power laser printing unit is not suitable for outer space processing due to its low electro-light conversion rate. On the other hand, low-power laser units, such as the one used in this study, cannot provide sufficient energy and fails to bond the formed single track and the baseplate effectively, as shown in Figure 4b. In this paper, a Joule heating source is introduced, which can remedy the energy shortage with only a single-laser heat source. It is known that, in a Joule heating source, electricity is directly converted into heat, without intermediate energy conversion, and its energy utilization rate can reach 100% [39]. The power supply provides a constant current flowing through the resistance between the conductive nozzle and the baseplate. Because the contact resistance between the wire and the baseplate is large and the current is constant, the largest amount of heat is generated in the contact area and the wire tip is melted first and fed into the molten pool which is generated by the laser heating. Moreover, the extremely hot metal wire increases the rate at which the metal absorbs the laser energy [40,41] and more energy can be put into the pool, thereby enhancing the energy utilization as a whole. As the hardness of the hot wire reduces and the bridging improves, the process of deposition becomes stable and smooth [42,43], thus achieving higher surface quality. During multi-layer deposition, using the low-power laser unit in continuous mode can ensure the formation of a shallow and steady molten pool [44], thereby reducing the quantity of the heat accumulated in subsequent layers. Therefore, the parameters are adjusted to control the heat input and heat accumulation in order to prevent collapse and deformation during AM.

### 4.2. Major Influential Factors on the Morphology of the Single-Track Parts

When laser power, Joule heating power and baseplate temperature are constant, the single-track parameters are correlated with the *K* and *V_M_* value. The relationships between them were determined by multiple regression analyses on nine sets of experiment data in Table 7. They are:

*H* = 10.7 + 1.08 ∗ V*_M_* + 249 ∗ *K* (5)

*W* = 276 − 0.549 ∗ V*_M_* + 51.6 ∗ *K* (6)

**Figure 10:**
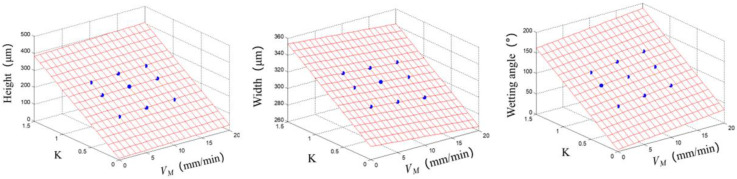
Multiple regression analysis for *H*, *W* and *α* of single-track versus *K* and *V_M_*.

*α* = 5.35 + 1.367 ∗ V*_M_* + 104 ∗ *K* (7)

as shown in Figure 10. From Equations (5)–(7) and Figure 10 it is clear that, the slower the baseplate is moving, the larger the amount of energy input per unit time, and the higher the temperature is in the printing area and the wire is melted more completely, resulting in a single-track section with larger width (*W*), smaller height (*H*), and smaller wetting angle, *α*. On the other hand, under the same conditions, when the parameter *K* becomes larger, the amount of wire to feed and to melt per unit time increases, and *H*, *W* and *α* increase linearly with *K*. Hence, *K* and *V_M_* exert significant effect on the regularity of the single-track dimensions and morphology.

### 4.3. Optimization of Thin-Walled Part Printing Parameters

For the proposed LJWF-DMD to be performed successfully, the input-power, wire-feeding speed (*V_W_*), baseplate moving speed (*V_M_*) and the baseplate temperature should be well-coordinated. Given a limited total power input, the parameters *K* and *V_M_* should be adjusted properly to ensure the evenness over total number of layers. It is also noted that inappropriate layer height (Δ*h*) could cause the print head to hit and even damage the thin-wall of the part in process, resulting in failure.

The scheme of parameter adjustment is created based on the heat transfer principles. Assuming that the wire, the baseplate with preheating plate are homogeneous, the Fourier law for heat transfer by conduction during the experimental process is modelled as follows:

(8) $$Q_{1}=-kA_{1}\frac{\partial T}{\partial x}$$

where *Q*_1_ is the power of heat transfer by conduction (W); *k* is the thermal conductivity (W·m^−1^·°C^−1^); and *A*_1_ is the heat transfer area (m^2^).

The thermal convection at the part surface satisfies Newton’s law of cooling:

(9) $$Q_{2}{=\mathrm{hA}}_{2}\left(T-T_{2}\right)$$

The radiation heat loss at the part surface satisfies the Stefan-Boltzmann law:

(10) $$Q_{3}=\varepsilon \sigma A_{2}\left(T^{4}-T_{2}^{4}\right)$$

where *Q*_2_ is the power of convective heat transfer (W); *h* is the coefficient of convective heat transfer (W·m^−2^·°C^−1^); *A*_2_ is the area of convective and radiation heat transfer (m^2^); *Q*_3_ is the power of radiative heat transfer (W); *σ* is the Stefan-Boltzmann constant; *ε* is the relative radiance; and *T*_2_ is the ambient temperature (°C).

**Figure 11:**
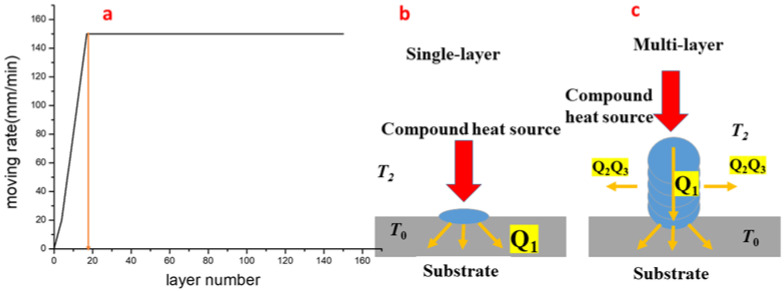
(**a**) Relationship between the moving speed at each layer of the thin-walled part. (**b**) Heat dissipation of the 1-st layer. (**c**) Heat dissipation of several layers.

In Equation (8), the heat transfer velocity is directly proportional to the heat transfer area and the temperature gradient. The energy provided by the baseplate pre-heating system increases the initial temperature (*T_0_*) of the thin-walled part printing area. Figure 11b shows that, at the first layer, the heat (*Q*_1_) is dissipated through conduction directly to the baseplate, a large metal sheet. As more and more layers are built up, the path of conductive heat, *Q*_1_, changes, dissipating downward, and the thermal resistivity of the thin-walled part increases along the height of the part, thus making it difficult for the laser heat to transfer, resulting in heat accumulation which causes the molten pool to become larger and even to collapse. Equations (9) and (10) show that as the surface area of the part increases in the process, convective and radiative heat transfer gradually take dominance over conduction. When a heat balance was reached between energy input and heat dissipation via conduction, convection and radiation, the dimensions of the molten pool settles at steady values, without increasing as more and more layers build up. To ensure the dimensional accuracy and surface roughness, the printing parameters must be adjusted. Among them, the easiest way to expedite the energy allocation and dimensional stability of the molten pool is changing *V_M_*. Experiments indicate that progressively adjusting *V_M_* layer by layer (Δ*h*) can yield desired results. Figure 11a shows the *V_M_* for each layer, with the thickness of the baseplate at 2 mm, Δ*h* 0.11 mm at *K* = 1 and 0.15 mm at *K* = 1.5, laser power of 50 W, Joule heating current of 10A, and baseplate temperature of 500 °C. The value of *V_M_* is increased progressively from beginning until the 17-th layer, and is not adjusted more afterwards. The dimensional accuracy and roughness of the specimen formed by the LJWF-DMD are shown in Figure 5, Figure 6 and Figure 7, indicating a desirable result can be obtained by optimizing crucial parameters, *V_M_*, Δ*h* and *K*. Although there are many other factors involved in the process that affect the temperature field distribution in the heat transfer system, it is feasible to adjust certain parameters to control the dimensions of the molten pool when heat balance is reached. Under heat balance, the thermal resistance is almost unchanged and thus the process parameters are adjusted more. To sum, optimizing energy allocation and adjusting crucial parameters can greatly increase the dimensional accuracy and roughness, and is expected to find wide practical applications.

## 5. Conclusions

A direct metal deposition additive manufacturing (DMD AM) with laser and Joule heating combination is proposed and studied experimentally. By using this method of LJWF-DMD, high quality of surface of the thin-walled parts can be achieved with low input-power. The dimensions and physical properties of single-track thin-walled parts were measured and tested. From the analysis and experiment results, the conclusions are as follows:

(1) Introducing a Joule heating source decreases the demand for laser power, and thus reduces the total printing power and heat accumulation and increases the surface quality. The maximum optical power during actual printing measured 50 W.
(2) Thin-walled parts formed by using the LJWF-DMD have aspect ratios as high as 40, uniform thickness and average deviation as low as approximately 0.017 mm. Thin-walled parts with 66° inclinations are built. No visible defects, such as pores and cracks, are observed on all the specimens.
(3) Progressively adjusting the parameters can improve the surface quality of the thin-walled parts. The surface roughness (*Ra*) below 5 μm is far smaller than that obtained using WF-based metal AM techniques and on a par with that obtained using SLM. The horizontal tensile strength is approximately 500 MPa; the elongation at fracture is 90%; even and fine dimples are observed on the fracture surface; the hardness is approximately 170 HV and is uniformly distributed at different heights; and the density is 99.37%. The LJWF-DMD is competent with the conventional processing techniques in terms of physical properties.
(4) Low surface roughness alleviates the burden of post-processing the formed parts. Using electrolyte-plasma processing can greatly reduce the specimen surface roughness, from 3.6 to 0.4 μm in ten minutes only.
(5) The proposed printing technique, LJWF-DMD, is expected to be applied in cases where only low-power laser is available while the required surface quality is higher, e.g., AM in outer space.

Overall, the thin-wall specimens fabricated by LJWF-DMD AM technique exhibit good surface quality and better density, hardness and mechanical properties. Future studies will base on multi-bead overlapping and complex structure process of LJWF-DMD AM. Additionally, the energy optimization strategy of multi-heat sources in the process by simulation can be considered.

## Figures and Tables

**Table 1:** Chemical Compositions of 316 L stainless steel.

Elements	Fe	Cr	Mn	Mo	Ni	Si	C
Wt%	64.447	17.3	1.74	2.66	13.1	0.73	0.023

**Table 2:** Characteristics of LJWF-DMD AM system.

Parameter	Value
Laser system type	Model YLPN-WELD-DEM0-2
Laser type	Ytterbium fiber laser
Laser mode	CW(continuous wave)
Emission wave length	1064 nm
Laser Max power	50 W
Laser incidence angle	50–80°
Wire feeding angle	30–60°
Min. beam diameter	80 μm
Maximum moving rate	300 mm/min
Maximum wire feed rate	600 mm/min
Wire feeding direction	Front
Wire diameter	0.3 mm
Shielding gas	Ar
Oxygen content	≤10 ppm
$K=\frac{V_{W}}{V_{M}}$	(1)

**Table 3:** The 150-layer thin-walled part thickness measurements (in mm), *K* = 1.

Number	1	2	3	4
upper	0.595	0.589	0.602	0.604
lower	0.568	0.573	0.563	0.557

**Table 4:** Surface roughness of LJWF-DMD AM thin-walled parts, mechanically measured.

*K*	Direction	*Ra* (μm)	*R_z_* (μm)
1	Horizontal	0.7	4.2
1	Horizontal	0.8	3.9
1	Horizontal	0.7	3.4
1	Vertical	2.3	12.1
1	Vertical	2.2	10.8
1	Vertical	2.4	12.1
1.5	Horizontal	2.0	11.6
1.5	Horizontal	1.4	12.6
1.5	Horizontal	2.3	9.9
1.5	Vertical	3.8	17.9
1.5	Vertical	3.3	17.3
1.5	Vertical	3.8	18.4

**Table 5:** Comparison of the proposed LJWF-DMD process with some research works from the literature.

Materials	Process	Surface Roughness	Reference
Powder	SLM	*Sa* (4–10 μm)	Imade, K. et al. (2018) [30]
	SLM	*Ra* (3–4 μm); *Rz* (16–20 μm)	Matras, A. (2020) [31]
	EBM	*Ra* (18–22 μm)	Borrelli, R. et al. (2019) [17]
Wire	WAAM	*Ra* (200 μm)	Xiong, J. et al. (2017) [32]
	FW-LMD	*Ra* (8–16 μm)	Muhammad, O.S. et al. (2019) [22]
	**LJWF-DMD**	*Ra* (3–5 μm); *Rz* (4–20 μm); *Sa* (4–8 μm)	**This study**

**Table 6:** Specimen images and surface roughness at different time points.

Time	0 min	2 min	4 min	6 min	8 min	10 min
Specimens	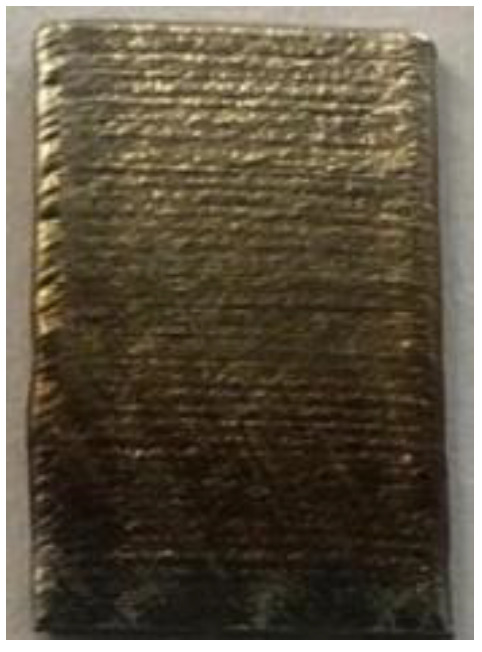	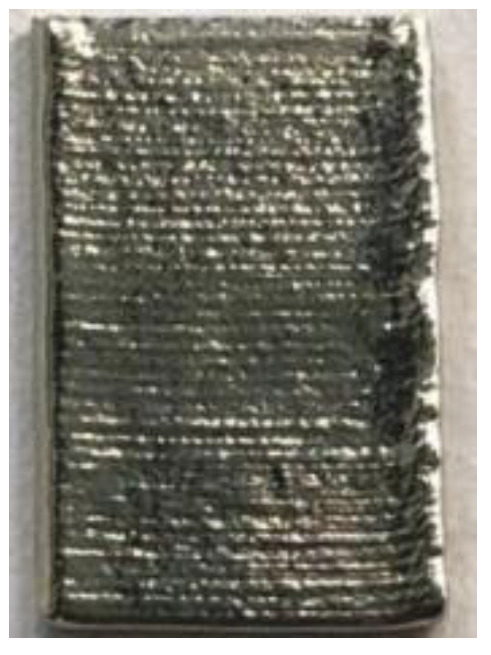	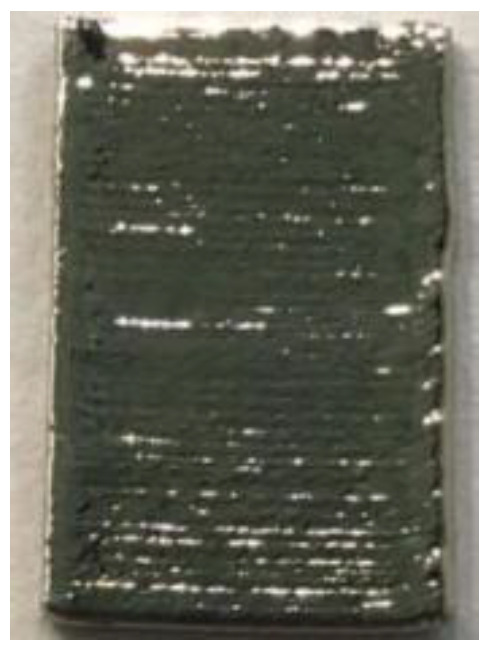	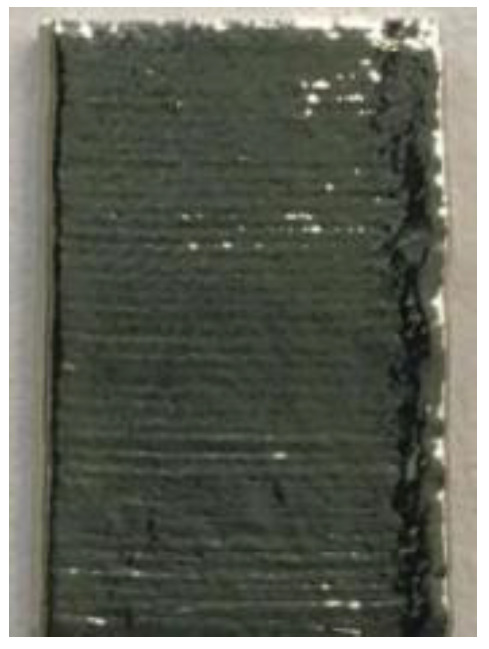	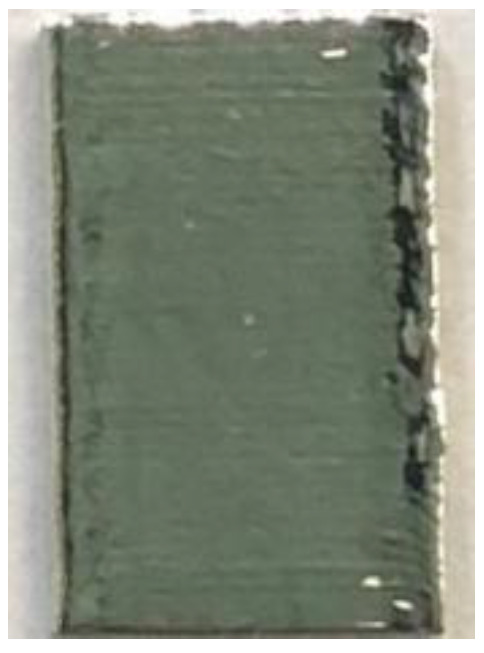	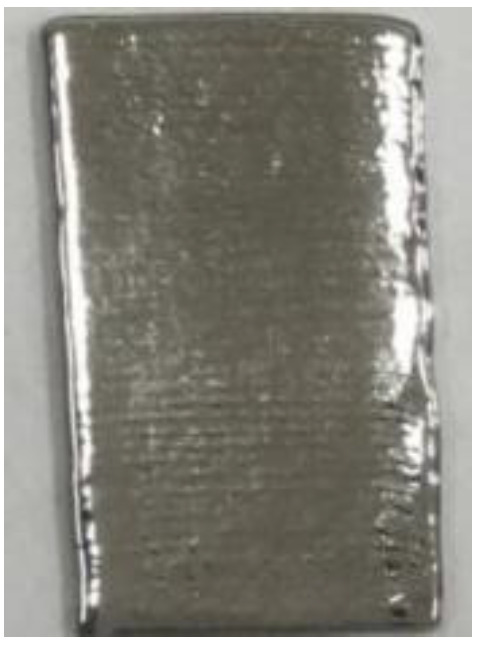
*Ra* (μm)	3.6	2.5	1.3	0.7	0.5	0.4

**Table 7:** Single-track process parameters: *H*, *W* and α.

Number	1	2	3	4	5	6	7	8	9
*K*	1	1	1	0.8	0.8	0.8	0.5	0.5	0.5
*V_M_* (mm/min)	5	10	15	5	10	15	5	10	15
*H* (μm)	262	271	272	218	224	228	137	147	150
*W* (μm)	325	323	321	314	312	309	300	297	294
*α* (°)	116	124	132	96	100	106	64	72	79

## Data Availability

The data presented in this study are available upon reasonable request from the corresponding author.
